# Treatment and outcomes for locally recurrent rectal cancer in Norway

**DOI:** 10.2340/1651-226X.2025.42991

**Published:** 2025-06-11

**Authors:** Ghazwan Al-Haidari, Arne M. Solbakken, Linn Merete Åsli, Eva Skovlund, Christine Undseth, Marianne Grønlie Guren

**Affiliations:** a Department of Oncology, Oslo University Hospital, Oslo, Norway; b Section of Abdominal Cancer Surgery, Department of Surgical Oncology, The Norwegian Radium Hospital, Oslo University Hospital, Oslo, Norway; c Department of Public Health and Nursing, Norwegian University of Science and Technology (NTNU), Trondheim, Norway; d Institute of Clinical Medicine, University of Oslo, Oslo, Norway

**Keywords:** Locally recurrent rectal cancer, surgical resection, radiotherapy, reirradiation, overall survival, multimodal treatment

## Abstract

**Background:**

The rate of locally recurrent rectal cancer (LRRC) in Norway has decreased due to advancements in surgical techniques and preoperative treatments. Despite this, LRRC continues to present significant morbidity and mortality challenges. This study aims to analyze survival outcomes following different treatment modalities for LRRC in Norway and assess the impact of changes in treatment strategies over time.

**Methods:**

This retrospective study utilized data from the Cancer Registry of Norway, focusing on patients with stage I-III primary rectal cancer treated between 1997 and 2016, who subsequently developed LRRC. Treatment modalities, including surgery, radiotherapy (RT), and re-irradiation (reRT), were analyzed, and the impact of various factors on overall survival (OS) was assessed.

**Results:**

Of the 13,480 patients who underwent surgery for rectal cancer, 827 (6.1%) developed LRRC. For all patients, the median survival from LRRC diagnosis was 18 months, with a 3-year OS of 29%. For patients who underwent surgical resection of LRRC, the 3-year OS was 55% for those who received pre-operative RT, 50% for those who received reRT, and 35% for those without any radiation therapy. For non-operated patients, 3-year OS rates were 22% with RT, 21% with reRT, and 15% for patients without radiation therapy. Patients diagnosed after 2006, patients with early-stage primary cancer, younger age (<75), extended recurrence interval, or well-differentiated tumors had better survival outcomes.

**Interpretation:**

This study describes the outcomes after multimodal treatment approaches for LRRC on a national level over a 20-year period. Patients who underwent surgical resection combined with RT or reRT had the best survival outcomes; however, this group represents a highly selected patient population.

## Introduction

The rate of locally recurrent rectal cancer (LRRC) has declined over the last decades due to the standardization of surgery with total mesorectal excision (TME) and the use of preoperative radiotherapy (RT) or chemoradiotherapy (CRT) for patients with locally advanced tumors [1–4]. In Norway, the local recurrence rate after primary resection is currently 4% [5]. Patients with recurrence have high morbidity, poor quality of life, and poor survival [6–8].

The mainstay of treatment for LRRC is surgery, with the aim of complete tumor resection (R0). This is challenging and often involves multivisceral resections in fibrotic tissue due to previous surgery. R0 resection is less common for LRRC than for primary tumors (55% vs. 80%) [9, 10] and many recurrent tumors are non-resectable [11]. In patients who have undergone surgery and achieved R0 resection, 5-year overall survival (OS) ranges from 35 to 60% [12–14], compared to 0–4% in non-resected patients [11, 14].

Preoperative RT **or** CRT are recommended for resectable LRRC [15]. In patients with previous pelvic radiation of the primary rectal tumor or other pelvic malignancies, reirradiation (reRT) is used. ReRT is feasible, and the primary objective is to increase the proportion of patients who will undergo R0 surgical resection, thereby improving local control and survival [16–18], but it has also been shown to provide effective symptomatic alleviation when used as a palliative treatment [19–21]. Several reRT regimens have been suggested. Hyperfractionated CRT (1.2–1.5 Gy twice daily) has been used to deliver high radiation doses (40.8–45 Gy) while minimizing the risk of late toxicity [22]. Once-daily reRT is also safe, effective, and provides symptom control [23].

In Norway, resectable LRRC is treated with CRT or reRT, followed by surgery [24]. In a previous institutional series, we found that reRT, especially when followed by surgery, was associated with favorable rates of resectability and survival [25]. Patients not eligible for resection may have symptom relief from palliative reRT. However, treatment may vary across health regions and with time. In Norway, curative treatment of LRRC, including surgery and reRT, is centralized to four specialized centers, while palliative treatment may be available in additional hospitals. We therefore sought to investigate treatment practice and outcomes in a national cohort of all patients with LRRC.

This study aimed to evaluate survival outcomes following different treatment modalities for LRRC across Norway over a 20-year period.

## Material and methods

### Registry data

The Cancer Registry of Norway (CRN) was established in 1951 [26]. Patients are registered with their unique national personal identification number, date of birth, sex, and county of residence, in addition to all cancer incidents. Disease-specific registries are thereafter linked to this main register, providing detailed information on treatment and outcome. In 1993, the Norwegian Rectal Cancer Registry (NRCR) was established for rectal cancer, registering the stage at diagnosis and treatment of the primary tumor. In 2007, the NRCR was extended to include colon cancer and more detailed information on pathology and treatment of metastases and was named the Norwegian Colorectal Cancer Registry (NCCR). In addition, information on RT, pathology reports, and updated survival status are provided.

### Patients

This study included all patients of primary rectal cancer stage I-III in the NRCR and NCCR who had undergone resection from January 1st, 1997 to December 31st, 2016, and who later developed LRRC. The CRN ensures high data quality through stringent registration practices primarily involving surgeons and pathologists, and follows up yearly with reminders to report data on locoregional recurrence [4]. Data on recurrence is thus considered highly reliable, as clinicians are required to report locoregional recurrences annually, regardless of the presence or timing of distant metastases. In contrast, data on metastases were deemed insufficient for analysis due to inconsistent reporting and lack of systematic follow-up from the CRN, which may result in incomplete documentation of whether metastases preceded or coincided with the recurrence. Date of birth, sex, county of residence, date of diagnosis, disease stage at primary diagnosis (retrieved from the pathology report of the surgical specimen), distance from anal verge, Carcinoembryonic Antigen (CEA), date of recurrence, details of RT delivered (for primary cancer, recurrence, or metastasis), surgery (including approach, type of rectal resection, and radicality of surgery), and survival data were obtained from CRN. Resection margins, including circumferential resection margin (CRM), allowed registration of resection status according to the residual tumor classification, with R0 defined as a tumor with > 1 mm resection margin, R1 ≤ 1mm, and R2 as a residual tumor after surgery [27]. The CRN lacked comprehensive information on chemotherapy; therefore, RT and reRT are reported, although they are typically administered concomitantly with 5-FU or capecitabine. Although the development and locations of distant metastases were documented, data quality and completeness were considered insufficient, and metastasis data were excluded from the survival analyses.

From 1997 through 2016, RT data were accessible. Date of RT, irradiated location, treatment intent, and RT dose were among the RT information obtained from each RT center and linked to the relevant cancer case. For operated patients, radiation therapy was defined as neoadjuvant if given 120 days or less prior to surgery, and adjuvant if given up to 90 days postoperatively. Survival was defined as the time from the date of the LRRC diagnosis to death.

### Radiotherapy for primary rectal cancer

The treatment of primary rectal cancer was based on national guidelines in Norway at the time [28]. Patients with T4 tumors or tumors near the mesorectal fascia (2–3 mm) were typically offered CRT prior to surgery, and the use of CRT increased during the study period [4]. The clinical target volume (CTV) was delineated in accordance with national guidelines [29]. RT was mostly given with 46.0 Gy in 2.0 Gy fractions to the CTV, with an additional 4.0 Gy to the gross tumor volume (GTV). Concomitant 5-fluorouracil (5-FU) or capecitabine was recommended. Some patients underwent short-course radiation therapy, that is 25.0 Gy in 5 fractions to the CTV; primarily older or frail patients [28, 30]. Conformal radiotherapy (3D-CRT) with a three-field setup (posterior and two lateral fields) was mostly used.

### Radiotherapy for local recurrence

For local recurrence, CRT, as described above, was recommended for previously non-irradiated patients, followed by surgery. For previously irradiated patients, indications for reRT, treatment volume, total dose, and fractionation schedule were less standardized and evolved over time. A margin ranging from 0.8 to 1.0 cm was added to the GTV to define the CTV, while elective regions were not included for re-irradiation (Re-RT). An additional margin of 0.7 to 1.0 cm or 0.5 to 1.0 cm was then added for the Planning Target Volume (PTV) or Internal Target Volume (ITV), respectively.

Earlier treatments utilized 3D-CRT, whereas later treatments employed intensity-modulated radiation therapy (IMRT) and, subsequently, volumetric arc therapy (VMAT). Total doses ranged from 30.0 Gy to 45.0 Gy and were frequently administered as hyperfractionated RT (1.2–1.5 Gy twice daily), though daily fractions of 2.0 Gy were also used. Concomitant capecitabine was recommended. However, the frequency of each RT technique (3D-CRT, IMRT, VMAT) could not be reliably quantified, as this information was inconsistently reported in the registry.

All patients with recurrence were assessed in multidisciplinary team (MDT) meetings, with decisions tailored to each case. Surgery was performed at expert referral centers, one in each of 4 health regions, and included pelvic exenteration if necessary to achieve complete tumor resection.

After radiation treatment, patients were either recommended surgery with resections of pelvic organs if deemed necessary for complete tumor resection or to further conservative treatment in a palliative setting.

The study was approved by the Regional Ethical Committee (2016/1970 REK Sør-Øst B) and by the Institutional Review Board.

### Statistics

Numbers and percentages (categorical variables) or medians and ranges (continuous variables) were used to describe patient characteristics (Table 1). The patient group was divided in an early (1997–2006) and a late (2007–2016) time period, reflecting two time periods with different primary tumor RT indications and recurrence rates, and also an improved data registration in the NCCR. OS, defined as the time from diagnosis of LRRC to death from any cause, was estimated by the Kaplan–Meier method. Patients still alive at the time of analysis (30 June 2017) were considered censored. OS was analyzed using Cox proportional hazards regression, with both unadjusted and adjusted hazard ratios (HRs) and 95% confidence intervals (CIs) presented in Table 2. Initially, unadjusted HRs were estimated for factors such as treatment of recurrence, time from primary diagnosis to recurrence, age at recurrence, primary tumor stage, and other relevant variables. To account for potential confounders, we then performed multivariable Cox regression, adjusting for all relevant covariates. Significance was set at *p* < 0.05.

**Table 1:** Tumor and treatment characteristics and treatment of patients with recurrent rectal cancer in the time periods 1997-2006 and 2007-2016 in Norway

		1997-2006 n=586	2007-2016 n=241	Total n=827
Primary tumor		N (%)	N (%)	N (%)
Sex	Male	345 (59)	160 (66)	505 (61)
	Female	241 (41)	81 (34)	322 (39)
Age, median (range) years		69 (29-93)	69 (30-90)	69 (29-93)
T and N-stadium (%)	pT1	9 (1.5)	13 (5.4)	22 (3)
	pT2	91 (16)	46 (19)	137 (17)
	pT3	411 (70)	144 (60)	555 (67)
	pT4	71 (12)	21 (9)	92 (11)
	Missing	3 (0.5)	17 (7)	20 (2.4)
	pN0	269 (46)	126 (52)	395 (48)
	pN1	156 (27)	57 (24)	213 (26)
	pN2	155 (27)	32 (13)	187 (23)
	Missing	3 (0.5)	26 (11)	29 (3.5)
Tumor grade (%)	High differentiation	19 (3.2)	18 (7.5)	37 (4.5)
	Moderate differentiation	450 (77)	166 (69)	616 (74.5)
	Poor differentiation	95 (16)	31 (13)	126 (15)
	Missing	22 (3.8)	26 (19.8)	48 (5.8)
Primary treatment	Neoadjuvant/adjuvant RT for primary rectal cancer	109 (19)	97 (40.2)	206 (24.9)
Recurrent tumor		N (%)	N (%)	N (%)
Metastasis at time of local recurrence		162 (28)	73 (30)	235 (28)
RT for recurrence		295 (50)	86 (36)	381 (46)
Dose of RT in Gy, median (SD)		50 (10.2)	50 (9.9)	50 (10.1)
				
reRT for recurrence		51 (9)	61 (25)	112 (14)
Dose of reRT in Gy, median (SD)		30 (10.6)	40.8 (8.8)	38.7 (9.96)
Total dose RT +ReRT, median (SD)		80 (15.4)	90.8 (15.6)	86.4 (15.5)
Operated for recurrence (%)		227 (39)	94 (39)	321 (39)
Resection margin after surgery for LRRC (%)	R0	139 (78)	38 (81)	179 (80)
	R1	36 (20)	9 (19)	45 (20)
	R2	3 (1.7)	0	3 (1.3)
	Missing	49	47	96
Operated for distant metastasis other than pelvis (%)		39 (7)	55 (23)	94 (11)

RT; radiotherapy, reRT; reirradiation, SD; standard deviation

**Table 2:** Overall survival

		N	3-year OS %	Unadjusted estimates	Adjusted estimates	
				HR	HR (CI 95%)	P value
Treatment of recurrence;						
	No treatment	245	15	1	1	
	RT+ Operation	187	55	0.32	0.32 (0.25-0.4)	<0.001
	reRT + Operation	33	50	0.47	0.47 (0.3-0.74)	<0.001
	Op with No RT/reRT	101	35	0.44	0.45 (0.33-0.58)	<0.001
	RT with No op	224	22	0.63	0.63 (0.56-0.78)	<0.001
	reRT with No op	33	21	0.91	0.9 (0.61-1.37)	0.64
Time from primary diagnosis to recurrence						
	≤20 months	433	24	1	1	
	>20 months	394	31	0.85	0.71 (0.61-0.84)	<0.001
Age at recurrence						
	< 50 years	37	35	0.41	0.48 (0.32-0.73)	0.003
	50-75 years	470	37	0.48	0.58 (0.49-0.70)	<0.001
	>75 years	320	13	1	1	
Primary cancer						
	1997-2006	586	29	1	1	
	2007-2016	241	38	0.78	0.70 (0.57-0.87)	<0.001
Primary tumor stage						
	T1-2	159	40	1	1	
	T3	555	26	1.44	1.32 (1.1-1.6)	0.001
	T4	92	15	1.96	1.48 (1.08-2.0)	0.01
Primary tumor N-stage						
	N0	359	36	1	1	
	N+	400	27	1.23	1.23 (1.04-1.44)	0.013
Tumor differentiation grade (No.)						
	Low (126)	126	17	1	1	
	Middle (616)	616	31	0.69	1.02 (0.54-1.9)	0.96
	High (37)	37	25	0.68	0.67 (0.44-1.03)	0.066
	Unknown (33)	33	18	0.61	1.1 (0.62-1.97)	0.061
Resection margin of the primary operation						
	R0	677	30	1	1	
	R1	129	13	1.65	1.36 (1.09-1.7)	0.006
	R2	3	0	1.83	2.4 (0.68-8.6)	0.17

Statistical analyses were performed using IBM SPSS statistics version 23.0.0.2.

## Results

**Figure 1:**
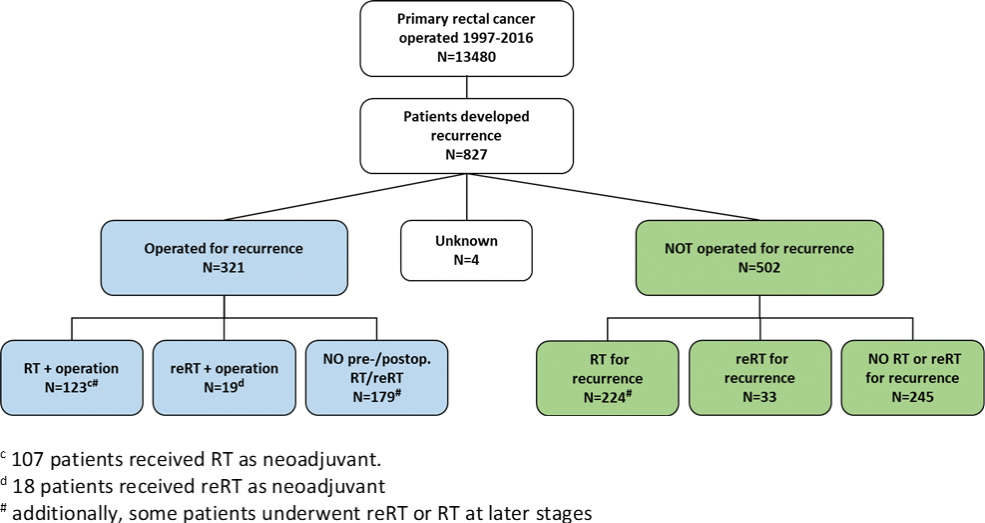
Flowchart of patients with recurrence of rectal cancer after primary surgery from 1997 to 2016.

From 1997 to 2016, 13,480 patients underwent major surgery for rectal cancer stages I–III (median 680 per year; range 581–761). A total of 827 patients (6.1%) developed LRRC, with or without metastases, and were included in the analysis (Figure 1). Of these, 586 (71%) occurred prior to 2007 and 241 (29%) subsequent to 2007. The incidence of LRRC showed a significant decline over the two decades: it was 8.5% in the earlier period (1997–2006), but decreased to 3% in the later period (2007–2016). The median age at diagnosis was 69 years (range: 29–93) and 505 patients (61%) were male (Table 1). For the primary tumor, neoadjuvant or adjuvant CRT/RT was delivered to 206 patients (25%), and the majority of patients had a pT3/T4 tumor. The median time from the initial diagnosis of rectal cancer to the LRRC was 20 months (range 1–114 months).

At the time of LRRC, 28% of patients also had metastatic disease. However, due to inconsistent reporting across the study period, this variable was not included in the survival analyses. Of all patients with LRRC, 321 patients (39%) underwent surgery, and in 227 of these, R-status was reported. A total of 179 (79%) of patients with available R-status achieved an R0 resection margin, 78% in the early period and 80% in the later period (Table 1). Among operated patients, 142 (44%) received neoadjuvant/adjuvant RT or reRT, whereas 179 (56%) did not (Figure 1). Of the 502 patients who did not undergo surgery for LRRC, 224 underwent RT, and 33 had reRT. The median radiation dose for patients who received reRT was 30 Gy prior to 2007 and 40.8 Gy after 2007. Accordingly, the median total dose was 80 Gy prior to 2007 and 90.8 Gy afterward (Table 1).

### Survival

The median OS from the time of recurrence was 18 months (range, 1–233), and the 3-year OS was 29–47% for the 321 patients operated for their recurrence, and 18% for the 502 patients not operated. Key factors associated with longer OS in LRRC patients were multimodal treatment, an interval of over 20 months between primary tumor diagnosis and recurrence, being younger than 75 years, a primary cancer stage of pT1–2, N0, highly differentiated tumors, and a primary cancer diagnosed from 2007 onward (Table 2).

**Figure 2:**
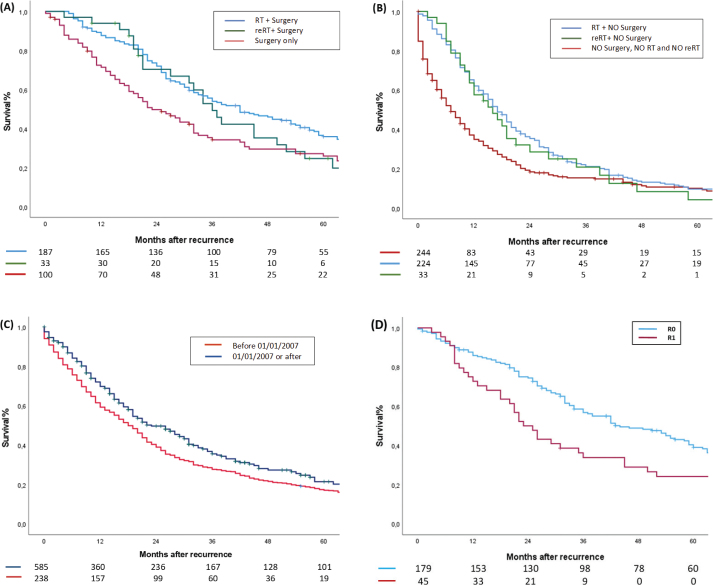
Overall survival after recurrence. (A) Survival of patients operated for recurrence. (B) Survival of patients not operated for recurrence. (C) Overall survival after recurrence prior to January 1, 2007 and afterward. (D) Survival of patients operated for recurrence with reported R-status (R0 and R1 only; R2 excluded due to low sample size). RT: primary radiotherapy; reRT: re-irradiation.

The type of treatment for LRRC had a significant effect on survival. For operated patients, the 3-year OS was 55% for those who received RT, 50% for those who received reRT, and 35% for those who did not receive radiation therapy before surgery (Figure 2A). For patients not operated on, the 3-year OS was 22% for those who received RT, 21% for reRT, and 15% for those who did not receive radiation for the LRRC (Figure 2B).

The 3-year OS was significantly higher for patients diagnosed with rectal cancer after 2006 (38% compared to patients diagnosed in earlier time period (29%) (Figure 2C).

**Figure 3:**
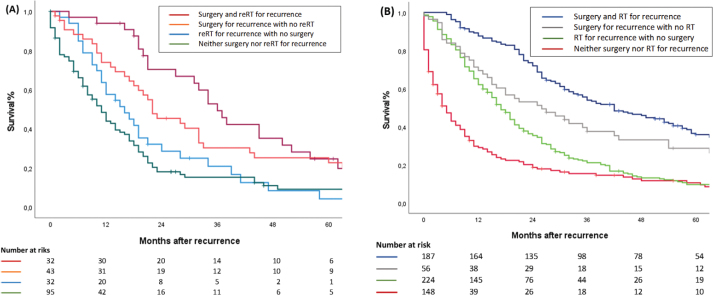
(A) Overall survival of locally recurrent rectal cancer (LRRC) patients who received neoadjuvant or adjuvant radiation therapy before surgery for the primary cancer. (B) Overall survival of LRRC patients who underwent primary surgery without additional radiation therapy.

A positive association was found between the treatment modalities used for primary cancer and LRRC, and OS. This association favored patients who underwent only surgical intervention for their primary cancer. In cases where patients had undergone RT for the primary tumor, the 3-year OS rates were 52% for those treated with reRT and surgery for LRRC, 30% for surgery alone for LRRC, 21% for reRT alone, and 15% for neither surgery nor reRT (Figure 3A). Conversely, for radio-naïve patients, the 3-year OS rates were 58% for RT and surgery for LRRC, 40% for surgery alone for LRRC, 24% for RT alone, and 18% for neither surgery nor RT (Figure 3B).

For patients operated on for LRRC with a reported R-status, the 3-year survival rate was significantly better for R0 compared to R1 and R2 (59, 37, and 0%, respectively). The hazard ratio (HR) was 1.8 (*p* = 0.001) for R1 and 3.8 (*p* = 0.024) for R2, in relation to R0 (Figure 2D).

## Discussion

The findings of this retrospective analysis provide informative perspectives on the management and prognoses of LRRC in Norway. The study underscores the significance of multimodal treatment strategies, especially the combination of surgical resection alongside RT or reRT, in enhancing survival rates among LRRC patients.

The study observes a significant reduction in the incidence of LRRC from 8.5% before 2007 to 3% after 2006. This decline is likely due to refinements in surgical methods, implementation of MRI in diagnostics, improvement in radiation therapy techniques, increased use of neoadjuvant CRT, and the introduction of structured MDT discussions in Norway during the study period – all contributing to more effective control of primary tumors and reduced local recurrence risks [4, 31]. These observations are supported by other studies, which have documented similar trends in rectal cancer treatment outcomes [32–34].

In Norway, surgery for LRRC has been centralized to four regional expert centers throughout the study period. This centralization likely contributed to the relatively high rate of R0 resections observed. In contrast, surgery for primary rectal cancer is also centralized to 27 hospitals, all regarded as high-volume centers across the country. The observed improvement in outcomes over time may therefore also reflect an indirect effect of centralization policies on the management of recurrent disease.

Our study supports the effectiveness of multimodal treatments in LRRC, particularly the combination of surgery with RT or reRT (55% 3-year OS for surgery with RT, 50% with reRT, 35% without radiation, 22% for RT alone, 21% for reRT, and 15% for no radiation) (Figures 2A and 2B). This is consistent with a review by Fadel et al. involving 974 patients, which found the highest R0 resection rates in those receiving neoadjuvant CRT, followed by neoadjuvant RT and adjuvant CRT (64.07% vs. 52.46% vs. 47.0%, respectively) [35]. In addition, this review reported the highest local control and OS rates in the neoadjuvant CRT group (5-year OS: neoadjuvant CRT at 34.92%, surgery only at 29.74%, neoadjuvant RT at 28.94%, adjuvant CRT at 20.67%). Our findings on the benefits of combining surgery with RT or reRT in LRRC are further supported by a systematic review of reRT in rectal cancer [20]. This review, involving 375 re-irradiated patients, supports the feasibility of reRT and its role in obtaining long-term survival in combination with surgery. It reports significantly better survival in resected patients (median OS: 39–60 months in resected patients; 12–16 months in palliative patients) and good symptomatic relief. These results collectively demonstrate the value of integrating multiple treatment modalities in LRRC management.

Our findings demonstrate better survival outcomes with factors such as early-stage primary cancer, younger age at diagnosis, well-differentiated tumors, and a longer interval between primary diagnosis and recurrence (Table 2). A recent study on colorectal cancer post-recurrence survival confirmed the impact of age, tumor stage, and histology on outcomes, with notably poorer results in older patients and stage III cancers. Mucinous tumors also predicted worse outcomes [36]. The poorer prognosis associated with early recurrence (within 20 months) has also been reported in another study (5-year OS – early recurrence 34.7%, late recurrence 78.8%; *p* < 0.001) [37].

R-status after operation for LRRC significantly influences survival outcomes, as evidenced by a 59% 3-year OS for patients with R0 vs. 31% for R1 resection in our cohort (Figure 2D). Hagemans et al. observed that younger LRRC patients undergoing surgery, particularly those with complete (R0) resections, had significantly better survival outcomes (5-year OS was 51% for R0-resections and 34% for R1-resections) [13]. This also aligns with prior research where the R0 resection rate was associated with a 50% 5-year OS [38]. This emphasizes the importance of resection margins as predictors of LRRC prognosis.

The survival benefit observed with RT or reRT in non-resectable cases suggests that RT plays an important role in local disease control, even when surgical options are not viable. This is corroborated by findings from a subgroup analysis in a systematic review, which included patients with LRRC who underwent reRT without surgery. Specifically, this subgroup demonstrated significant OS rates at 1-, 2-, and 3-year intervals, reported as 63.5, 34.2, and 23.8%, respectively [19]. Comparatively, our findings indicate a 3-year OS of 22% with RT, 21% with reRT, and 15% without radiation. Similar results have been reported in single-center experiences [25, 39]. These results highlight the potential of RT to effectively manage and extend survival in non-operated LRRC cases.

Stereotactic RT for LRRC has not been used in Norway during this time period. However, emerging evidence suggests that it may be a treatment alternative for selected patients [40]. Furthermore, the role of chemotherapy in this setting is not yet fully understood. Although total neoadjuvant treatment (TNT) has a role in primary rectal cancer, it is not yet standard for LRRC in Norway, however studies are ongoing. Current national guidelines focus on CRT or reRT prior to surgery, but TNT is being explored in clinical trials such as PelvEx II and GRECCAR 15, and may influence future treatment protocols. The ongoing PelvEx II trial is investigating the potential role of chemotherapy before RT or ReRT and surgery in patients with LRRC [41]. Additionally, the results of the GRECCAR 15 trial are awaited, where patients with LRRC are randomized to receive either chemotherapy and reRT or chemotherapy alone before surgery [42]. These and other studies are expected to inform future treatment decisions for this patient group.

Although this is a nationwide study, several limitations should be considered. As with any retrospective analysis, the study is subject to the limitations of historical data collection, including potential biases and missing data. This is particularly evident for R-status, which was available for only 227 out of 321 cases (71%). In addition, data on metastatic disease at the time of LRRC was incomplete and, therefore, not analyzed. This represents a significant limitation, as metastatic disease plays a critical role in treatment decisions and significantly impacts survival outcomes. Although local recurrences are consistently registered, some cases with prior or synchronous metastases may have been misclassified due to less complete metastasis reporting, potentially leading to minor underreporting or misclassification of recurrence patterns.

The absence of data on patient comorbidities and quality of life limits the ability to fully assess the factors influencing treatment selection and outcomes. Comorbidities can significantly impact treatment tolerability and OS, and the lack of quality-of-life data prevents a comprehensive evaluation of the long-term impact of multimodal treatments on patient well-being.

The study lacks comprehensive information on chemotherapy, which is a key component of advanced rectal cancer treatment. Future studies incorporating detailed chemotherapy data could provide a more complete view of the treatment landscape [41].

Quality of life and functional outcomes should be investigated in future studies, as they are important aspects of patient-centered care.

Prospective studies, potentially incorporating newer treatment modalities and systemic therapies, are needed to validate these findings and guide future treatment algorithms.

## Conclusions

This study underscores the significance of multimodal treatment approaches in managing LRRC, with the best survival outcomes obtained in patients who were treated with RT or Re-RT and surgery. Future research should incorporate chemotherapy data, quality of life assessments, and standardization of treatment protocols.

## Data Availability

The data used in this study were obtained from the Cancer Registry of Norway. Due to privacy regulations and data protection policies, the datasets are not publicly available. Access to the data may be granted upon reasonable request and with permission from the Cancer Registry of Norway and relevant ethical approval.
